# The Application of Polymer Inclusion Membranes for the Removal of Emerging Contaminants and Synthetic Dyes from Aqueous Solutions—A Mini Review

**DOI:** 10.3390/membranes13020132

**Published:** 2023-01-19

**Authors:** Małgorzata A. Kaczorowska, Daria Bożejewicz, Katarzyna Witt

**Affiliations:** Faculty of Chemical Technology and Engineering, Bydgoszcz University of Science and Technology, 3 Seminaryjna Street, PL 85326 Bydgoszcz, Poland

**Keywords:** emerging contaminants, pharmaceuticals, pesticides, synthetic dyes, polymer inclusion membrane, separation processes

## Abstract

Pollution of the environment, including water resources, is currently one of the greatest challenges due to emerging new contaminants of anthropogenic origin. Of particular concern are emerging organic pollutants such as pharmaceuticals, endocrine disruptors, and pesticides, but also other industrial pollutants, for example, synthetic dyes. The growing demand for environmentally friendly and economical methods of removing emerging contaminants and synthetic dyes from wastewater resulted in increased interest in the possibility of using techniques based on the application of polymer inclusion membranes (PIMs) for this purpose. PIM-based techniques are promising methods for eliminating emerging contaminants and synthetic dyes from aqueous solutions, including wastewater, due to high efficiency, membranes versatility, ease/low cost of preparation, and high selectivity. This review describes the latest developments related to the removal of various emerging contaminants and synthetic dyes from aqueous solutions using PIMs over the past few years, with particular emphasis on research aimed at increasing the effectiveness and selectivity of PIMs, which may contribute to wider use of these methods in the future.

## 1. Introduction

The term “emerging contaminants” (ECs) refers to synthetic or naturally occurring chemical compounds (and microorganisms) that have not been commonly monitored in the environment, many of which have been discovered recently [1]. The group of ECs is diverse and includes a variety of chemicals, such as, for example, pharmaceuticals, hormones (animal and human), personal care products, detergents (household and industrial), perfluoroalkyl and polyfluoroalkyl substances (PFASs), pesticides, industrial chemicals, and microplastics. Substances belonging to the ECs category can be classified according to a variety of criteria, e.g., based on their structure, size, properties, or functions. In the case of the criterion of the size and structure of ECs, two main categories were distinguished: chemicals of emerging concern and particles of emerging concern, wherein the second group includes only engineered nanoparticles and microplastics [2]. The division of ECs due to their functions is not simple because such substances can be used for various purposes in different products. For example, PFASs containing aliphatic carbon backbones with the side chain hydrogens substituted by fluorines are used in consumer goods such as surfactants, firefighting foams, alkaline cleaners, carpets, non-sticky pans, room polishers, shampoos, fume resistants, photo films, semiconductors, food packaging, pesticides, denture cleaners, and masks [3]. In principle, ECs may adversely affect ecological conditions and human health; however, as they may undergo various transformations (e.g., degradation processes) when introduced into the environment, it is not easy to determine their overall impact on living organisms and research in this area is still being conducted [1,4,5]. Many reports related to the analysis of different types of environmental samples (e.g., surface water and groundwater, soil) have confirmed the increasing presence of emerging contaminants and products of their transformations [6]. The increase in the amount of these substances in the environment results from their widespread use around the world. For example, pharmaceuticals and related compounds, i.e., active pharmaceutical ingredients (APIs), are consumed in large quantities, often without a prescription, consequently getting into the water cycle [7]. APIs found in wastewater include a vast variety of substances, among others, painkillers and anti-inflammatory drugs, antibiotics, hypoglycemic drugs, anti-ulcer drugs, anti-epilepsy drugs, etc. [8], and also narcotic substances such as cocaine, barbiturates, methadone, amphetamines, opiates, heroin, etc. [9]. Various persistent and environmentally hazardous anthropogenic PFASs have been detected not only in surface waters, soils, and biosolids (by-products in the sludge treatment process) but also in human tissues and blood serum [3,10,11]. Due to PFASs potential adverse effects on human health, the United States Environmental Protection Agency has set the combined concentration of per- and polyfluoroalkyl substances in drinking water at 70 µg/L as the lifelong health advisory level [12].

Pesticides are also one of the most common groups of pollutants found in the environment because the steadily increasing use of these compounds in agricultural practices (e.g., to destroy unwanted vegetation) has resulted in the occurrence of residues of these chemicals and their metabolites in groundwater, wastewater, and soil [13]. Pesticides have been proven by many researchers as toxic substances to humans and the environment. For example, chronic exposure to herbicides causes cardiovascular problems, retinal degeneration, some muscle degeneration, and cancer in humans [14]. Due to the properties of pesticides and the diversity of compounds belonging to this group of ECs, many countries, including the EU, have set limits for individual and total pesticides in treated drinking water, respectively (e.g., the EU Drinking Water Directive) [15].

The presence of emerging contaminants (e.g., pharmaceuticals and pesticides) in the water environment is a significant problem due to their toxicity. Traditional technologies for removing these compounds from wastewater (e.g., coagulation, adsorption, advanced oxidation) have disadvantages such as, for example, excessive and toxic sludge production and high production costs. Additionally, conventional wastewater treatment plants are unable to completely remove many of ECs; therefore, new effective methods of wastewater treatment are systematically sought [16,17,18].

The group of chemical compounds classified as synthetic dyes is also complex, but its largest part is made up of azo dyes [19]. Synthetic dyes, due to their properties (e.g., relatively simple and inexpensive synthesis, stability, resistance to light and oxidizing agents), are widely used for dyeing different products in various industries, e.g., in the textile and paper industries, for dyeing leather and other materials. However, the widespread use of synthetic dyes is also associated with serious environmental risks, especially to aquatic ecosystems. These compounds are usually resistant to recycling and can persist in the environment for a long time, potentially posing a threat to living organisms. To remove them from aqueous solutions (e.g., from industrial wastewater), various methods are used (such as adsorption, oxidation, coagulation, precipitation, ion exchange, or methods based on the use of microorganisms) [19,20]; however, each of these methods has certain limitations, which is why new, more efficient and eco-friendly methods of removing synthetic dyes from wastewater are being sought.

In recent years, there has been increased interest in the possibility of using various types of membranes to remove ECs and synthetic dyes from wastewater. In principle, membrane-based processes are considered more environmentally friendly than many traditional methods of removing these pollutants [21,22]. One type of liquid membranes (LMs) are polymer inclusion membranes, containing a polymer matrix, carrier, and most often a plasticizer, the use of which enables simultaneous extraction and re-extraction. Moreover, compared to other LMs, PIMs are characterized by better stability, longer lifetime, selectivity, and flexibility properties [23].

This mini-review presents an overview of the latest achievements in the use of polymer inclusion membranes for the removal of synthetic dyes and selected emerging contaminants (pharmaceuticals, endocrine-disrupting compounds, and pesticides) from aqueous solutions, with particular emphasis on the achievements of the last five years. It also contains information on the effectiveness and most important limitations in the application of the methods developed. Attention was focused on these compounds due to their systematically increasing consumption and the threat they pose to the environment.

## 2. Polymer Inclusion Membranes—Short Characteristics

Various contaminants can be removed from aqueous solutions using membrane techniques, e.g., with polymer inclusion membranes which have been successfully used, among others, to separate metal ions [24,25,26] and dyes [27,28,29] and different pesticides from aqueous matrices [30]. PIMs were also applied for the remediation of pharmaceuticals and endocrine-disrupting substances from water systems [16].

A membrane is a semi-permeable mass transport barrier between two phases (liquid or gaseous). It is essential that the individual components of the mixture (said mass) be separated and move at different speeds. Membrane methods are used, e.g., for gas separation and water purification, filtration, and separation. Such applications are possible thanks to the simple concept behind them, the modularity of the solution, low energy costs, ease of scaling up, and a positive impact on the environment. If membranes are viewed as semi-permeable phase separators, the traditional concept of a membrane as a polymeric film can be extended to include liquids. These membranes are called Liquid Membranes. The membrane system, in this case, consists of an organic liquid that is immiscible neither with the feeding (donor) phase nor with the receiving (acceptor) phase. The membrane is, in this case, a semi-permeable barrier between the phases, which are mostly aqueous solutions. Transport through liquid membranes includes both liquid-liquid extraction and membrane separation processes [31]. The commonly accepted transport mechanism across liquid membranes is diffusion. The components diffuse through the membrane due to the concentration gradient. The efficiency and selectivity of transport through the LM can be significantly increased by introducing a complex substance (carrier) into the liquid membrane. The carrier in the LM reacts reversibly with the substance in the donor phase to form a complex.

Solution components can be transported across the membrane in several ways. There are four possible ion transfer mechanisms:

(a) Simple transport;
(b) Assisted transport;
(c) Coupled counter-transport;
(d) Coupled transport co-transport [31].

Direct transport is one of the forms of simple transport and is defined as the phenomenon of the transfer of compounds caused by solubility in a liquid membrane. The process is continued until the substance concentrations in the feed and receiving phases are equalized. The transferred substance does not react chemically with the membrane components; it remains in the same form in both phases. Another form of simple transport occurs when the transferred substance becomes a substrate and reacts with a compound contained in the receiving phase. Then, the concentration of the substance to be removed will not reach equilibrium, and the transport will continue until the complete transition of molecules from the feeding phase takes place [31].

Assisted transport in liquid membranes is one of the most important applications of supramolecular chemistry. It can occur by partitioning, complexation, or diffusion reaction. The substance from the feed phase is complexed by a carrier (ionophore) contained in the membrane and released to the receiving phase. The conveyor increases the speed of transport and ensures a significant selectivity of the process. It lasts until the concentrations on both sides of the LM are equal or until the substance is completely removed from the feeding phase. Assisted and simple transport can occur simultaneously.

The synthesis and use of various chemical compounds as carriers in liquid membranes have been the subject of intensive research by scientists over the last few decades [31,32].

Counter-transport, on the other hand, is transport that takes place against the concentration gradient. It involves the transfer of substance A from the feed phase to the receiving phase by means of a carrier, with the simultaneous transfer of substance B (usually a hydrogen ion) in the opposite direction. When the concentrations of substance B are equalized, the process stops. Appropriate planning of the process, including the selection of concentrations, allows for the complete extraction of substance A from the feed phase.

In membrane co-transport, a liquid substance is co-transported with an accompanying component. The type and properties of the latter affect the parameters of the process that takes place until the concentrations are equalized. The speed of transport is regulated by the concentration of the accompanying substance. Co-transport is the most common form of transport across liquid membranes [33].

**Scheme 1:**
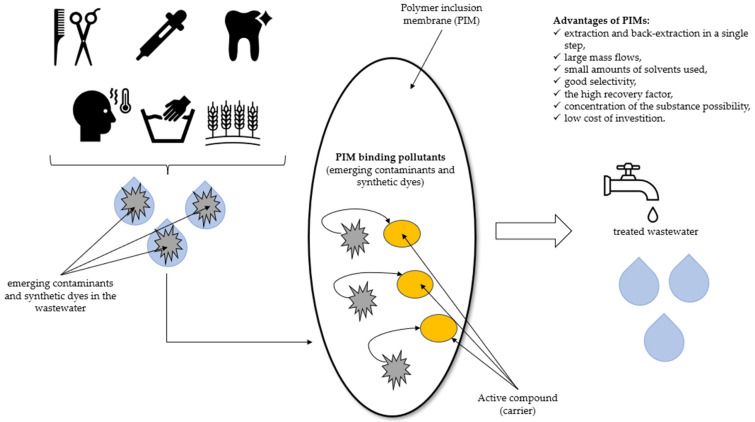
The advantages of PIMs used for the removal of emerging contaminants and synthetic dyes.

PIMs belong to the category of Supported Liquid Membranes (SLM) [33,34]. The matrix for the construction of the SLM membrane is usually made of plastics, e.g., poly(vinyl chloride), polypropylene, polyamides, modified cellulose, etc. [35]. The main problem of SLM technology is stability. This includes, e.g., the chemical stability of the carrier as well as the mechanical resistance of the porous substrate. In order to improve the stability parameters, newer and better systems are being created. For example, PIMs are created by pouring a solution obtained by mixing a polymer, an organic phase (carrier), and a so-called plasticizer. The plasticizer is added to the mixture to reduce the hardness and strength of the membrane while increasing its flexibility [31]. PIMs have many advantages, as shown in Scheme 1.

## 3. Applications of PIMs for the Removal of Selected Emerging Contaminants

### 3.1. Removal of Pharmaceuticals and Endocrine-Disrupting Compounds by PIMs

Pharmaceuticals are a large group of various chemical compounds used, among others, in human health protection (e.g., anti-inflammatory drugs, antibiotics, painkillers, beta-blockers, etc.) and animal treatment (various veterinary drugs), in food preservation, and in agriculture. Their consumption is systematically growing, and it is expected that it will continue to grow due to, inter alia, the extension of the average life expectancy of people [16]. Due to the structural diversity of pharmaceuticals and the fact that they are usually found in small amounts in environmental and wastewater samples, their determination often requires the use of techniques allowing for analyte isolation and enrichment for trace analysis, which complicates the entire process and increases its costs. Removing small amounts of hazardous substances from the environment is also not easy. The processes of separation of pharmaceuticals based on the use of membranes, such as polymer inclusion membranes, not only allow the removal of even small amounts of contaminants from complex matrices with high efficiency and selectivity but also fit into the trend of the so-called “green analytical chemistry” (consumption of small amounts of chemical reagents, reduction of the use of toxic solvents, the possibility of multiple uses of membranes) [36]. Recently, PIMs have been used to remove from aqueous solutions one of the most widely utilized pharmaceuticals, ibuprofen (IBF), a non-steroidal anti-inflammatory drug, intended, inter alia, for lowering fever, pain relief and applied in the treatment of rheumatoid arthritis [8]. There is a need to develop an effective method of removing ibuprofen from the environment because it has been shown that prolonged accumulation of this compound can adversely affect the hormonal balance in the aquatic systems [8,16].

Moulahcene et al. [37] prepared novel polymer inclusion membranes containing insoluble poly(β-cyclodextrin (β-CD) polymer as a carrier, poly(vinyl chloride) as a base polymer, and dibuthylphtalate (DBP) as a plasticizer, and used them for the elimination of ibuprofen and progesterone from model aqueous samples. They found that the effectiveness of ibuprofen extraction depends on the content of β-cyclodextrin in the membrane, the agitation of analyzed samples, and reaction pH. The highest efficiency of the ibuprofen removal process (approx. 50%) was observed in an acidic environment (pH~2.0). However, based on these results, it is not possible to clearly determine the suitability of β-CD-based PIMs for removing ibuprofen from real samples because wastewater samples contain many other components that can potentially affect the membrane process. Ahmad et al. [8] reported that modification of PIMs with graphene oxide (GO) and small amounts of Aliquat 336 (methyltrioctylammonium chloride) as carrier allows for the removal of ibuprofen from model aqueous solutions. They reported that GO + Aliquat 336—doped polymer inclusion membranes made of PVC as base polymer without adding any plasticizer allow for the extraction of ibuprofen with an efficiency of ~84%, ~83%, and ~77% at a feed solution pH of 10, 6, and 2, respectively. Although obtained results indicate a high potential for ibuprofen extraction using GO-doped PIM better than those obtained with PIM containing poly(β-cyclodextrin [37]. Similar results (recoveries of analyzed pharmaceuticals in the range of 81–34%) were received by Román-Hidalgo et al. [38]. They used PIMs containing Aliquat 336 and cellulose triacetate (CTA) as base polymer, which were the support of 1-octanol liquid membranes in electromembrane extraction (EME) for the simultaneous removal of four non-steroidal anti-inflammatory drugs (such as salicylic acid (SA), ketoprofen (KET), naproxen (NAP), and ibuprofen) and four highly polar acidic drugs (such as anthranilic acid, nicotinic acid, amoxicillin, and hippuric acid). They also reported that the tested PIM can be applied for the extraction of these chemicals from real urine samples.

Removal of antibiotics from wastewater is also of great importance because, when introduced into the environment, they can have a toxic effect on flora and fauna, and in addition, they can potentially increase the resistance of microorganisms to antibiotics. Traditional methods of wastewater treatment do not allow the complete removal of these compounds, which is why research is systematically conducted on the possibility of using other techniques [39]. Polymer inclusion membranes have been used during the last few years for the preconcentration of antibiotics in order to allow the determination of trace amounts of these substances in environmental water samples. For example, Garcia-Rodriguez et al. [40] successfully utilized PIMs containing CTA or PVC as base polymers, Aliquat 336 as a carrier, and 2-nitrophenyloctyl ether (2-NPOE) as a plasticizer for the preconcentration of aqueous samples of sulfonamides (SAs) and tetracyclines (TCs). Garcia-Rodriguez et al. [41] also developed a passive sampler containing a polymer inclusion membrane (CTA/Aliquat 336/2-NPOE) for monitoring sulfamethoxazole (SMX) (one of the antibiotics most frequently detected in wastewater treatment plants, which can be harmful to living organisms) in water systems. They reported that the designed passive sampler minimized the influence of the flow pattern of the aquatic medium monitored, which is a significant property because the flow pattern of water cannot be controlled and possibly have an influence on the reliability of passive sampling data collection. PIMs were also used in novel methods based on a combination of various techniques. Recently, it has been reported that the hollow fiber polymer inclusion membrane liquid-phase microextraction (HF-PIM-LPME) methods, based on the use of PIMs and being a modification of the conventional HF-SLM-LPME method (Hollow Fiber Supported Liquid Membrane Liquid-Phase Microextraction), in which SLM was replaced with PIM, can be successfully used for the preconcentration of pharmaceutically active compounds in aqueous solutions. It was shown that HF-PIM-LPME is more stable compared to HF-SLM-LPME and is characterized by good selectivity for the analyzed pharmaceuticals [42]. Olasupo et al. [43] reported the efficient removal of sulfamethoxazole antibiotics from aquatic samples by utilization of an electromembrane extraction process based on polymer inclusion membranes comprised of the CTA polymer, Aliquat 336 carrier and the plasticizer dioctyl phthalate (DOP). Since sulfamethoxazole extraction using only PIM was insufficient (after 40 h of extraction at pH 9, transport efficiency was 82%), in order to improve the transport of the antibiotics across the membrane, they used electromembrane extraction and examined the impact of electric voltage on the efficiency of the extraction process. The use of a voltage of 50 V allowed for the complete removal of sulfamethoxazole from the aqueous solution (transport efficiency of 100%). Moreover, the membrane was used in 12 consecutive cycles, and only a slight decrease in extraction efficiency was observed, which confirms its stability. Formulated PIMs (CTA/Aliquat 336/DOP) were also successfully used to preconcentrate sulfamethoxazole from real environmental samples. However, it was reported that applying a voltage was essential to achieving the total recovery of this antibiotic. Olasupo et al. [43] obtained better results in comparison with results obtained by Román-Hidalgo et al. [38], who used the same PIM-EME method and the same PIM recovered 81% of analyzed pharmaceuticals.

Proper selection of all membrane components and thorough optimization of all experimental conditions enables the use of PIMs alone for the recovery of various antibiotics from aqueous solutions with satisfactory efficiency. Recently, polymer inclusion membranes with PVC and CTA base polymers and different carriers, such as bis-2-(ethylhexyl) phosphate (B2EHP) and Aliquat 336, have been applied for ciprofloxacin (CIP) removal from model aqueous solutions and real river water and wastewater samples. Ciprofloxacin has been shown to be persistent in the environment. In addition, it is present in various environmental media, which is due to the fact that this antibiotic is widely used and conventional wastewater treatment methods do not allow for its complete removal. The results of performed experiments show that at optimum conditions (proper selection of pH, receiving phase composition), the removal efficiency of 99.2% was noted using PVC/B2EHP PIM and 95% with the use of CTA/B2EHP membrane. The application of Aliquat 336 as the carrier allowed for obtaining the highest removal efficiency of 60%. The authors concluded that the formulated PIMs with B2EHP can be potentially considered a suitable alternative for the removal of CIP from the aquatic environment [44]. In turn, Olasupo et al. [45] applied PIMs containing PVC as the base polymer, B2EHP as the carrier, and DOP as the plasticizer for the removal of ciprofloxacin from the aquatic system and optimized the experimental conditions (i.e., pH of the source solution, source solution concentration, carrier concentration, the concentration of the receiving solution, and extraction time) using multivariate analysis of half-fractional factorial design. They reported that PIMs with the composition of PVC 45%: B2EHP 25%: DOP 30% allowed for almost complete removal of CIP, wherein removal efficiency strongly depended on pH (>99% at pH = 6, 90% at pH = 12, and 85% at pH = 7). The method was also successfully applied to remove ciprofloxacin from real environmental wastewater and river samples with an average percentage removal efficiency of >97%. Based on the results of the use of PVC/B2EHP/DOP PIMs for CIP removal, it can be assumed that this method may also be potentially useful in the future for the removal of other pharmaceuticals from aqueous systems.

Polymer inclusion membranes may also in the future play an important role in the removal of chemical compounds from aqueous solutions that are substrates for the synthesis of traditionally used drugs. An example of such a compound is salicylic acid, which is used in the synthesis of aspirin and glyburide (and is also a metabolite of aspirin). Additionally, it is also used as an analgesic and antipyretic drug and prevents platelet aggregation. It has been reported that salicylic acid has been detected not only in sewage but also in drinking water and is characterized by high ecological toxicity ([46] and references therein). Therefore, the development of an effective method of removing this compound from complex matrices is extremely important for environmental protection. Recently, Meng et al. [47] examined the suitability of a polymer inclusion membrane containing PVC as a base polymer and *N,N*-bis(1-methylheptanyl)acetamide (N503) as a carrier for the separation of salicylic acid and two phenols from aqueous solutions. They analyzed the influence of carrier concentration and membrane thickness on the morphology, structure, and mass transfer stability of formulated PIMs. Their results have shown that polymer inclusion membranes had a good mass transfer capability for salicylic acid and p-nitrophenol (PNP) through a hydrogen transfer mechanism in weakly acidic environments with a properly selected receiving phase. The mass transfer of salicylic acid exhibited the characteristics of a fixed-site hopping mechanism. Moreover, at pH = 6, the phenol and p-nitrophenol coexisting with salicylic acid in the analyzed samples were selectively removed because, in such conditions, salicylic acid molecules formed intramolecular hydrogen bonds and were not transported effectively.

Efficient methods are also systematically sought to remove phenolic compounds from aqueous solutions (e.g., sewage), classified as potential endocrine-disrupting chemicals (EDCs), such as bisphenol and octylphenol. These compounds cause exogenous endocrine disruption in the neurological, reproductive, and immune systems, even in very small amounts, and are classified as highly toxic pollutants [48]. Balahouane et al. [49] used polymer inclusion membranes with calix[4]resorcin arene derivatives as the carrier, CTA as the polymer matrix, and 2-NPOE as a plasticizer to eliminate bisphenol A (BPA) from synthetic wastewater (model solutions containing bisphenol A) and to determine the process parameters that most affect its performance. They found that the efficiency of the bisphenol A removal process using PIMs was affected mainly by the concentration of membrane components, stirring rate, pH of the feed phase, and thickness of the membrane. Under optimal conditions of the membrane process, about 90% of bisphenol A was transported from the feed phase to the receiving phase, wherein PIM was an efficient tool in bisphenol A removal only when this contaminant was present in the solution in an undissociated state. The prepared membranes were also successfully used in three successive cycles of removal of this compound. All presented examples of the application of polymer inclusion membranes for the removal of various pharmaceuticals and endocrine disruptors show that with the (qualitatively and quantitatively) proper selection of membrane components and carrying out the membrane process in optimal conditions (for each pollutant removed, experimentally determined), separation based on utilization of PIMs is a promising technology for removing such organic contaminants from their aqueous solutions. As noted by Olasupo et al. [16], PIM “has a great potential for the remediation of pharmaceuticals and endocrine disruptors in the aquatic system, due to its versatility, ease/low cost of preparation and high contaminant selectivity”.

**Figure 1:**
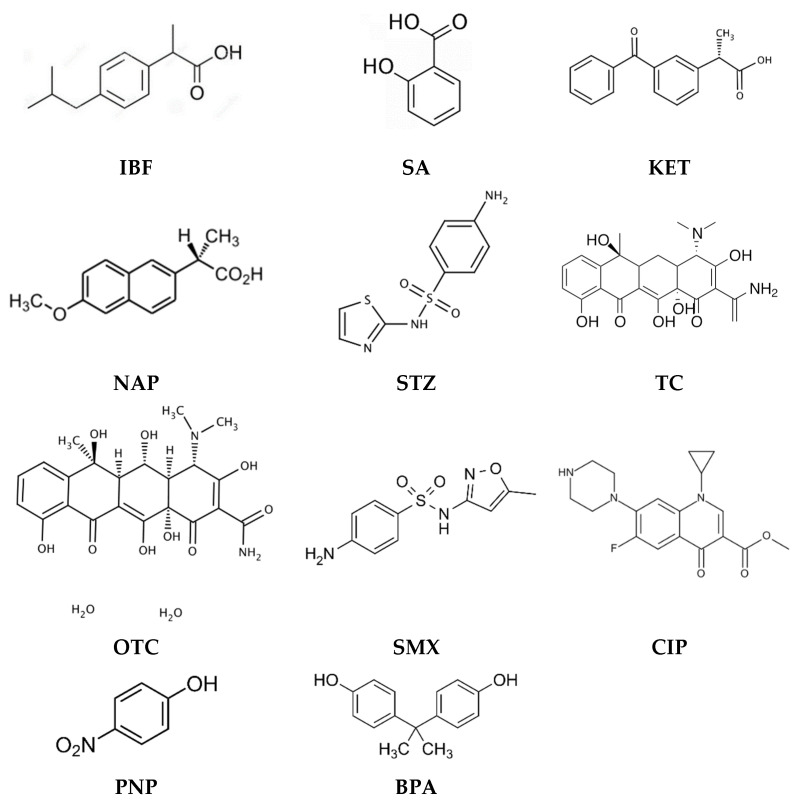
Structures of pharmaceuticals and endocrine-disrupting compounds removed by PIMs.

Table 1 shows examples of the use of PIMs for the removal of pharmaceuticals and endocrine-disrupting compounds, such as IBF, SA, KET, NAP, sulphathiazole (STZ), TC, oxytetracyline (OTC), SMX, and also CIP, PNP, and BPA (Figure 1).

### 3.2. Removal of Pesticides Using PIMs

**Figure 2:**
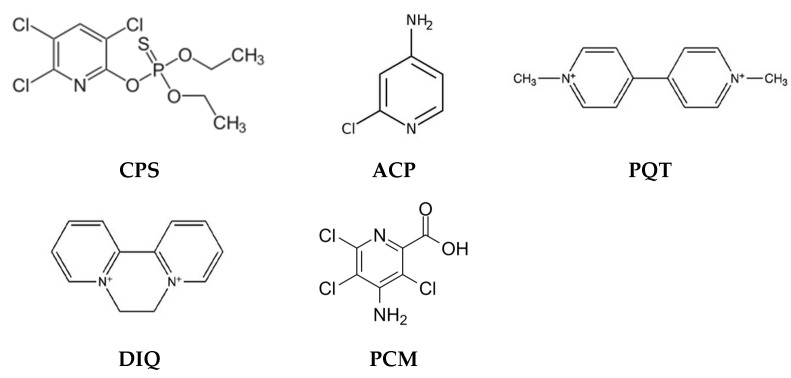
Structures of pesticides removed by PIMs.

According to the World Health Organization, pesticides are chemical compounds used to fight parasites, including insects, rodents, fungi, and weeds. A lot of pesticides released into the environment have been defined as toxic, persistent, bioaccumulative, and harmful to the health of the entire ecosystem, including humans [50]. Depending on the type of pesticide and the nature of the environmental sample, various methods are used to determine the content of these compounds, to identify them, and finally to remove pesticides (e.g., solvent extraction (SE), Solid-Phase Extraction (SPE), Solid-Phase Microextraction (SPME), High-Resolution Mass Spectrometry (HRMS), chromatography-based methods, etc.), however, all these solutions have certain limitations (e.g., SE—consumption of large amounts of toxic solvents) [50,51]. Therefore, other solutions are sought. Methods based on the use of PIMs are becoming increasingly popular because they are environmentally friendly, efficient, and selective and allow the removal of even small amounts of pesticides from aqueous solutions. Examples of removal of various pesticides, e.g., chlorpyrifos (CPS), 4-amino-2-chloropyridine (ACP), paraquat (PQT), diquat (DIQ), and picloram herbicides (4-amino-3,5,6-trichloro-2-pyridinecarboxylic acid) (PCM) (Figure 2) by methods based on the application of PIMs, are shown in Table 2.

For example, Vera et al. [52] used a new extraction phase based on a polymer inclusion membrane containing cellulose triacetate (70%) and nitrophenyl octyl ether (30%) for the detection of three different pesticides: chlorpyrifos, diazinon, and cyprodinil in natural water samples. They evaluated the main factors affecting the extraction efficiency (i.e., membrane composition, stirring speed, extraction, and elution time) and reported that PIM-assisted extraction allows pesticide determination in the range of 50–1000 mg/L with good linearity and high recoveries (85% and higher, depending on pesticide). They found that CTA/NPOE PIM-assisted extraction is especially useful for the detection of chlorpyrifos in river waters (RF = 93%). Mwakalesi and Potter [53] applied polymer inclusion membranes containing anacardic acid (AA) obtained from cashew nut shells as an acidic carrier, CTA as a polymer matrix, 2-NPOE as a plasticizer and dodecanol as a modifier for the extraction of 4-amino-2-chloropyridine, paraquat and diquat. These compounds can be treated as representative target solutes of organic pesticide residues. They reported that PIM with optimal composition (i.e., 30 wt% CTA, 40 wt% NPOE, 10 wt% AA, 20 wt% dodecanol) has an extraction performance similar to polymer inclusion membrane containing commercially available synthetic bis-(2-ethylhexyl) phosphoric acid instead of AA. Additionally, AA containing PIM has also been successfully used for ACP, paraquat, and diquat removal from real environmental water samples. The developed method based on the use of PIMs with AA originating from natural sources (e.g., plants and plant waste) is part of the trend of using eco-friendly methods for the treatment of polluted waters. Another example confirming that polymer inclusion membranes with a properly selected composition in optimal conditions are excellent tools for removing pesticides from aqueous solutions are the results of research conducted by Mwakalesi and Potter [54]. They applied PIMs containing CTA polymer, NPOE plasticizer, and Aliquat 336 carrier to remove picloram (4-amino-3,5,6-trichloro-2-pyridinecarboxylic acid), which is an herbicide used to control the growth of woody plants. Because of the poor adsorption of picloram by soils and its high leaching properties, this compound often occurs in aqueous environmental samples. Due to its adverse effect on aquatic organisms and animals, there is a need to develop safe (for living organisms) and efficient methods to remove picloram from aquatic systems. Results of performed experiments demonstrate that CTA/NPOE/Aliquat 336 PIMs with an optimized concentration of ingredients and type and concentration of stripping reagent showed good rates of flux and transport efficiency for picloram in both non-competitive and competitive transport studies. Moreover, it has been reported that this membrane can be used in a passive sampling device to recover picloram (at the maximum concentration of 500 μg/L) from a complex matrix of natural water. In principle, in the last decade, in the environmental monitoring of waters for the presence of various types of pesticides, polymer inclusion membranes have played an important role and are increasingly used for, inter alia, passive sampling, sample pre-treatment, and preconcentration and also for removing these compounds from aquatic mediums [56]. There were also attempts to use polymer inclusion membranes in combination with other techniques to develop efficient methods to remove hazardous pesticides from water systems. For example, Mamat et al. [46] developed a new, more complex method for removing cationic and anionic herbicides from aqueous solutions based on the use of modified polymer inclusion membranes, namely electric-field driven extraction in which a bubbleless electrode has been integrated with electromembrane extraction across hollow polymer inclusion membranes (HPIMs). New hollow membranes based on PIMs were employed because they can be successfully used in electromembrane extraction, and, in principle, they improve extraction efficiency and reproducibility compared to traditionally used supported liquid membranes. In performed experiments, two types of HPIMs containing CTA as a polymer matrix and tris(2-ethylhexyl)phosphate as a plasticizer were used: with di-(2-ethylhexyl)phosphoric acid as an anionic carrier or Aliquat 336 as a cationic carrier. The electromembrane extraction based on the utilization of HPIMs made it possible to simultaneously determine both cationic quaternary ammonium and anionic chlorophenoxy acetic acid herbicides from river water samples with an extraction efficiency ranging from 99.1% to 100%. Based on the obtained results, the authors predict that the proposed approach may be potentially applicable in the future to remove other (both organic and inorganic) polar ions from aqueous solutions [57]. However, the use of an appropriate method based on membrane techniques to remove various pollutants, including pesticides, from the aquatic environment is not easy due to the chemical diversity of the chemical compounds removed, the complexity of the matrix in which they occur, different physicochemical properties of membranes and the wide range of parameters affecting the efficiency and selectivity of the separation processes [58].

## 4. Use of PIMs for Removal of Synthetic Dyes

**Figure 3:**
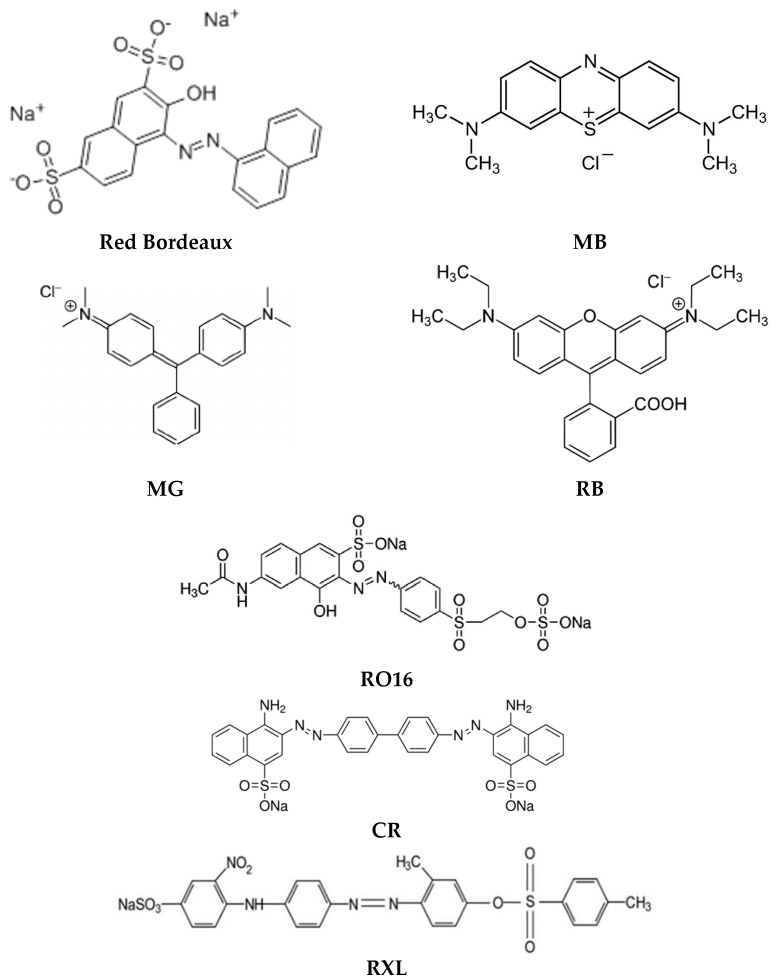
Structures of synthetic dyes removed by PIMs.

Water pollution associated with the large-scale use of synthetic dyes, e.g., in the textile industry, is one of the most pressing environmental problems in the world. Synthetic dyes introduced into wastewater may hinder the operation of the sewage treatment plant, and introduced into the environment may be harmful to food chain species and aquatic organisms. At the same time, not only dyes can pose an environmental threat, but also products of their transformation, e.g., as a result of hydrolysis of some of these compounds, poisonous aromatic amines may be formed [59]. Due to the need to remove such contaminants from wastewater, new environmentally friendly methods with high efficiency but also low costs and easy implementation are systematically sought. The examples presented in this chapter (and in the simplified version in Table 3) clearly confirm that polymer inclusion membranes are well suited for the removal of various (both cationic and anionic) synthetic dyes from aqueous solutions, as evidenced by the high efficiency of the processes and the simplicity of membrane preparation and experimentation. Figure 3 shows the structures of acidic dyes (red Bordeaux, Yellow Erionyl (RXL), Congo Red (CR)), basic dyes (Methylene Blue (MB), Malachite Green (MG), Rhodamine B (RB)), and also reactive orange dyes (RO16).

For example, Salima et al. [60] used PIMs containing CTA as the base polymer, 2-nitrophenyl octyl ether as a plasticizer, and Aliquat 336 as a carrier for removing two types of acid dyes: red Bordeaux acid and yellow Erionyl from aqueous solution. To determine the optimal conditions for conducting the membrane extraction process, they analyzed the influence of such factors as the pH of the aqueous solution, the initial concentration of dyes in the feed solution and of Aliquat 336 in the membrane, and the stirring speed. They reported the extraction of 99% of analyzed synthetic dyes under optimized experimental conditions. Minhas et al. [29] applied an ester derivative of calix[4]arene (EDC) as a carrier in polymer inclusion membranes consisting of CTA as a base polymer and 2-NPOE as a plasticizer and used the prepared membranes for extraction of cationic synthetic dyes (methylene blue, basic red, and Nile blue) from aqueous solutions. After optimization of various experimental parameters (pH of the donor and the acceptor phases, temperature, stirring speed, and concentration of membrane components and dyes in feed solutions), PIMs enabled the extraction of about 90% of dyes. Moreover, the extraction efficiency of the PIMs remained similar after ten consecutive cycles. That, as noted by the authors, “demonstrates membranes reusability potential in context of economical industrial applications”. Ling and Suah [61] reported, based on the results of performed experiments, that PIMs can be applied successfully for the removal of synthetic dyes from both model and real wastewater solutions. They used polymer inclusion membranes containing PVC polymer, B2EHP extractant, and dioctyl phthalate plasticizer for the removal of malachite green dye and found that in optimal experimental conditions, the average extraction efficiency achieved was >98% for model solutions and >96% for wastewater samples. Results of experiments performed by Amin et al. [62] have also shown that polymer inclusion membranes are also suitable for the simultaneous extraction of two basic synthetic dyes, such as malachite green and methylene blue, from aqueous solutions. They used a batch reactor consisting of a polymer inclusion membrane composed of PVC as the base polymer, B2EHP as an extractant, and DOP as the plasticizer and reported that extraction efficiency was higher than 97% for the mixture of malachite green and methylene blue within 4 h of the extraction process. The results are similar to those obtained in previously described research [61] and further works [23,63,64]. Mustafa et al. [63] prepared 3-(aminopropyl)triethoxysilane (APTES) enhanced CuO nanoparticles integrated polymer inclusion membranes and used them for the removal of the methylene blue dye (model solutions) via transportation from the feed phase to the strip phase. They reported that after the optimization of experimental conditions (pH of the feed/receiving phases, stirring speed, time, the concentration of the carrier in the membrane, and concentration of the dye), a dye transportation efficiency of 97% was achieved. Studies on the development of efficient PIMs for removing synthetic dyes also refer to the possibility of using polymers other than CTA/PVC as a membrane matrix. Methylene blue and rhodamine B (cationic dyes) were also removed with comparable effect during facilitated transport on PIM (D2EHPA-PIM) [64]. The adsorption removal efficiencies under the optimal conditions reached about 93% and 97% for MB and RB, respectively. On the other hand, different extraction values were observed for MB (E% = 18.7%) and for RB (E% = 82.4%) during the process at pH 2. Desorption of both dyes from the membrane was achieved using acidic aqueous solutions, and a desorption ratio of up to 90% was obtained. The dye transport was elucidated using mass transfer analysis, where relatively high values of the initial fluxes (J_0_) were found: 41.57 and 18.74 μmol·m^2^·s^−1^ for MB and RB, respectively. Soo et al. [23] used PVDF-co-HFP as the base polymer for the formulation of PIM with B2EHP as a carrier and DOP as the plasticizer and used this membrane for the extraction of cationic dye malachite green from model aqueous solutions. With optimal composition in optimal experimental conditions, the formulated PIM was able to remove more than 97% of the synthetic dye. These results may potentially influence further directions of the development of PIMs because CTA/PVC-based PIMs usually demonstrate lower performance in long extraction periods, and PVDF-co-HFP has higher stability than other base polymers. Moreover, as demonstrated by Soo et al., it has high extraction and transport efficiency for malachite green synthetic dye. Recently, Benosmane et al. [65] reported that the application of PIMs consisting of CTA polymer, 2-NPOE plasticizer, and calix[4]resorcin arene (RC8) carrier allows the efficient removal of methylene blue dye from model solution as synthetic aqueous wastewater, wherein the carrier and plasticizer content in the membrane strongly influence the properties of PIMs. Abdul-Halim et al. [66] used PIM made from cellulose triacetate and Aliquat 336 to extract Congo red from an aqueous solution. They improved some of the parameters, such as Aliquat 336 concentration, pH of extraction solution, the concentration of Congo red, the temperature of the process, and stirring speed which had an impact on the dye removal. Finally, 87% of Congo red was successfully removed after 24 h by using CTA-PIM with 50 wt.% Aliquat 336 at the optimum conditions: pH = 2, temperature 30 °C, and stirring speed of 150 rpm, respectively Gunasegaran et al. [67] removed the reactive orange 16 (RO16) dye by PIM consisting of poly(vinylidene fluoride-co-hexafluoropropylene) (PVDF-Co-HFP) as a base polymer, and Aliquat 336 as a carrier. The optimum extraction of RO16 dye was found in an initial dye concentration of 10 mg/L in 4 h time at pH 2 with an agitation speed of 500 rpm at room temperature using PIM with 9% carrier content. The highest percentage of removal of RO16 dye was 99.62%. Results of experiments performed by Ravi et al. [27], who applied PIMs consisting of PVDF-Co-HFP as a polymer matrix and Aliquat 336 as a carrier for the removal of the dye reactive orange 16 from aqueous solutions, confirm the suitability of this co-polymer for the development of membranes intended for the removal of synthetic dyes. In optimal experimental conditions, the extraction efficiency of reactive orange 16 was almost complete (99.62%). The presented examples of the use of PIMs containing various carriers/polymers/plasticizers for the removal of different synthetic dyes confirm the high efficiency of these methods (extraction efficiency in most cases was well over 90%) and suggest that they can potentially be used on a wider scale in the future, e.g., for the treatment of industrial wastewater from the textile industry. However, further research is needed, especially in the field of membrane stability (the possibility of their multiple uses) and in the development of techniques for their regeneration.

## 5. Concluding Remarks and Future Perspectives

In recent years, scientists have been particularly interested in developing effective and environmentally safe methods of removing hazardous pollutants, such as emerging contaminants and synthetic dyes, from aqueous solutions [68,69,70,71,72]. The environmental threat from these pollutants is primarily due to economic growth. The pharmaceutical, cosmetic, textile, and many other industries generate a large amount of often toxic wastewater, which according to European law, must be properly treated. In some cases, the toxic compounds introduced into the water ecosystem can have serious acute effects on the organisms exposed to them [73]. Emerging contaminants (e.g., pharmaceuticals and pesticides) and synthetic dyes can be removed from aqueous solutions during various separation processes such as adsorption, solvent extraction, bioremediation, etc. Table 4 presents sample information on the methods used to remove specific emerging contaminants, such as pharmaceuticals and pesticides from various aqueous mediums, and the results achieved by their applications. Comparing the results described in Table 1 with the results obtained using various PIMs to remove these contaminants from aqueous solutions (described in the text), it can be clearly stated that in many cases, polymer inclusion membranes are much more efficient (e.g., in comparison to solvent extraction, adsorption, precipitation methods). In the case of comparable performance of PIMs and other methods, the advantages of PIMs (i.e., the ease of PIMs formulation, the possibility of using various chemical compounds for this purpose, low consumption of toxic solvents, the possibility of multiple use of membranes after regeneration, relatively low costs of PIMs separation and safety for the environment) may have a key, positive impact on the decision which of the solutions are more beneficial.

In conclusion, this article presents examples of the application of methods based on the use of polymer inclusion membranes in the last five years to remove selected emerging contaminants and synthetic dyes from aqueous solutions (model and real wastewater samples). Separation methods based on the application of PIMs have great potential and can probably be widely used in the future for the effective removal of such pollutants because they are economical and environmentally friendly. However, the application of PIMs to remove pharmaceuticals, pesticides, and synthetic dyes from wastewater also has some limitations. In order for the membrane process to be carried out with the desired efficiency, it is necessary to determine the proper composition of the membranes (qualitative and quantitative) and the optimal conditions for the membrane process (for each type of pollutant to be removed and each type of wastewater treated). It has been shown that a variety of chemical compounds can be used to formulate PIMs, both as a polymer matrix (e.g., CTA and PVC) or pollutants carriers (e.g., D2EHPA, Aliquat 336, N503, calix[4]resorcin arene). In addition, various modifications to PIMs can be introduced, e.g., the addition of different plasticizers and formulation of membranes containing two different carriers. Polymer inclusion membranes are also increasingly used in combination with other methods (e.g., EME). Since the membrane process is influenced by many factors (in addition to membrane composition and feed solution/wastewater composition, the composition of the receiving phase, pH, temperature, etc., play an important role), it is usually time-consuming to determine all the optimal conditions. Moreover, the efficiency of PIMs decreases after some time, so it is necessary to develop a fast and effective method for their regeneration or to change membranes frequently, which also affects the time needed to treat wastewater and the cost of the process. Solving problems related to the fouling of membranes will certainly contribute to their wider use in the future to remove various pollutants from the aquatic environment.

## Figures and Tables

**Table 1:** Examples of removal of pharmaceuticals and endocrine-disrupting compounds by methods based on utilization of PIMs.

The Composition of PIM	Additional Information	Removal Efficiency	References
PVC/β-CD/DBP	Acidic environment (pH 2)	50% of IBF	[37]
PVC/Aliquat 336 + GO	Various feed solutions: at pH 10, at pH 6, and at pH 2	~84% of IBF ~83% of IBF ~77% of IBF	[8]
CTA/Aliquat 336	Posterior HPLC separation with diode array detection	34–81% SA, KET, NAP, and IBF	[38]
CTA/Aliquat 336/2-NPOE	The feed solution was at pH 9, and the stripping solution contained 1.0 M NaCl	55% of SP, 75% of STZ, and 100% of TC, and OTC	[40]
CTA/Aliquat 336/2-NPOE	Receiving solution was 2.0 M NaCl	SMX	[41]
CTA/Aliquat 336/DOP	After 40 h of extraction at pH 9	82% of SMX	[43]
PVC/B2EHP	Extraction at pH 6	99.2% of CIP	[44]
CTA/B2EHP		95% of CIP	
PVC/Aliquat 336		60% of CIP	
PVC/B2EHP/DOP	An environmental: at pH 6, At pH 12 and 7, and the environmental wastewater and river samples	90% of CIP 85% of CIP >97% of CIP	[45]
PVC/N503	The hydrogen bond mechanism at pH 6	7.54 mg/g PNP and 8.88 mg/g SA	[47]
calix[4]resorcin arene	Separation at pH 4 and stirring at 600 rpm	90% of BPA	[49]

**Table 2:** Examples of removal of pesticides by PIMs.

The Composition of PIM	Additional Information	Removal Efficiency	References
CTA/NPOE	GC-MS prior PIM	93% of CPS	[52]
CTA/AA/2-NPOE/DDL	An initial flux of 364 (±16) × 10^−8^ mol m^−2^ s^−1^ for transport of ACP	ACP, PQT, and DIQ	[53]
CTA/Aliquat 336/2-NPOE	Receiving solution was 0.25 M NaCl	95% of PCM	[54]
	EME-HPIM	From 99.1% to 100% of cationic quaternary ammonium and anionic chlorophenoxy acetic acid herbicides	[55]

**Table 3:** Examples of removal of synthetic dyes by various PIMs.

The Composition of PIM	Additional Information	Removal Efficiency	References
CTA/Aliquat 336/2-NPOE	pH (2–9) of the aqueous solution, the concentration of extractant in the membrane (5–30 μmol/cm^2^), initial dye concentration (50–250 ppm), and the stirring speed (250–400 rpm)	99% of red Bordeaux acid and RXL	[60]
CTA/EDC/2-NPOE	EDC forms inclusion complex with cationic dyes having 1:1 M ratio	~90% of MB	[29]
PVC/B2EHP/DOP	The average extraction efficiency was >98% for MG concentration of 20–80 mg/L	>98 of MG from model solution and >96% of MG for wastewater samples	[61]
PVC/B2EHP/DOP	Receiving solution was 1.0 M HNO_3_, and 4 h of extraction process (with magnetic stirring). The feed solution was MG, MB, and the mixture of both dyes (all volumes are set to 100 mL)	>97% of MB and MG	[62]
PIM/APTES-CuO	APTES-modified CuO nanoparticles were integrated with PIM. The PIMs were fabricated with the diffusion-induced phase inversion technique	97% of MB	[63]
CTA/D2EHPA/2-NPOE	The feed phase was 20 mg/L MB or RB at pH 6.0. The receiving phase was 1.0 M HNO_3_ or 1.0 M H_2_SO_4_ in the case of the MB or RB transport, respectively	93% of MB and 97% of RB	[64]
PVDF-co-HFP/B2EHP/DOP	Feed phase was 120 mL of 10 mg/L of MG, and receiving phase was 1.0 M HNO_3_	97% of MG	[65]
CTA/RC8/2-NPOE	Feed solution was 100 mg/L of MB, and the receiving solution was 0.3 M HCl (pH 11)	72.55% of MB	[66]
CTA/Aliquat 336	At pH 2, with temperature and stirring speed of 30 °C and 150 rpm	87% of CR	[67]
PVDF-co-HFP/Aliquat 336	Feed solution was 10 mg/L of RO16, and receiving solution was 1.0 M HCl	99.62% of RO16	[68]
PVDF-co-HFP/Aliquat 336	Feed solution was 10 mg/L of RO16, and receiving solution was solution of HCl	99.62% of RO16	[27]

**Table 4:** Examples of using different methods to remove selected emerging contaminants from aqueous solutions.

Method	Active Compound	Contamination	Removal Efficiency	References
Adsorption	Mixed-linker Zr-MOFs	DIC	2.04 mmol/g	[74]
Adsorption	Active carbon	TC	477.1 mg/g	[75]
Adsorption	Graphene	ATN, CB, CIP, DIC, GFZ, and IBF	>90%	[76]
Adsorption	Magnetic nanoparticles: Cu-BTC@Fe_3_O_4_	DIC, IBF, CIP, and OFX	IBF 13.38, DIC 12.47, CIP 12.35, OFX 11.14 mg/g	[77]
Adsorption	CDP	Pesticides	22.5%	[78]
Electro membrane extraction	HPIM 1: (CTA + TEHP + DEHPA) HPIM 2: (CTA + TEHP + Aliquat336)	Herbicides	99.1–100%	[55]
Extraction	Covalent organic frameworks (COFs)	Pesticides	>80%	[79]
Dispersive solid phase extraction	EMR-Lipid: mixture of ethyl acetate and acetonitrile	Pesticides from the oil matrix	>95%	[80]
Solvent extraction	Bio-sourced ionic liquids	Pesticides	>95%	[81]
Bioremediation	Microalgae	Parabens	Close to 100%	[82]
Co-precipitation over activated carbon	MAAC	CIP	81.97 mg/g	[83]
Adsorption	Activated carbons from argan nutshells	DIC Caffeine	126 mg/g 210 mg/g	[84]

## Data Availability

Not applicable.

## Abbreviations

β-CD	Poly(β-cyclodextrin)
AA	Anacardic Acid
ACP	4-Amino-2-chloropyridine
Aliquat 336	Trioctylmethylammonium chloride
APIs	Active Pharmaceutical Ingredients
APTES	3-(Aminopropyl)triethoxysilane
ATN	Atenolol
BPA	Bisphenol A
B2EHPA or B2EHPA	Bis-2-(ethylhexyl)phosphate
*4-tert*-BP	4-Tert-butylphenol
CB	Carbamazepine
CDP	Cross-linked cyclodextrin polymer
CIP	Ciprofloxacin
COFs	Covalent organic frameworks
CPS	Chlorpyrifos
CR	Congo red
CTA	Cellulose Triacetate
DDL	Dodecanol
DES	Desipramine
DIC	Diclofenac
DIQ	Diquat
DOP	Dioctyl Phthalate
D2EHPA	Di(2-ethylhexyl)phosphoric acid
EDC	calix[4]arene
ECs	Emerging Contaminants
EDCs	Endocrine-Disrupting Chemicals
EME	Electro Membrane Extraction
GC-MS	The gas chromatography and mass spectrometry detection
GFZ	Gemfibrozil
GO	Graphene Oxide
HF-PIM-LPME	Hollow Fiber Polymer Inclusion Membrane Liquid-Phase Microextraction
HPIMs	Hollow Polymer Inclusion Membranes
HF-SLM-LPME	Hollow Fiber Supported Liquid Membrane Liquid-Phase Microextraction
HRMS	High-Resolution Mass Spectrometry
KET	Ketoprofen
IBF	Ibuprofen
IMI	Imipramine
LM	Liquid Membrane
MAAC	Magnetic activated carbon
MB	Methylene Blue
MG	Malachite Green
NAP	Naproxen
NOR	Nortriptyline
N503	*N,N*-bis(1-methylheptanyl)acetamide
2-NPOE	2-Nitrophenyloctyl ether
OFX	Ofloxacin
OTC	Oxytetracycline
PCM	Picloram herbicides (4-amino-3,5,6-trichloro-2-pyridinecarboxylic acid)
PIM	Polymer Inclusion Membrane
PNP	P-nitrophenol
PFASs	Perfluoroalkyl and Polyfluoroalkyl Substances
PQT	Paraquat
PVC	Poly(vinyl chloride)
PVDF-co-HFP	Poly(vinylidene fluoride-co-hexafluoropropylene)
RB	Rhodamine B
RC8	calix[4]resorcinarene
RO16	Reactive Orange 16 dye
RXL	Yellow Erionyl (acidic dye)
SA	Salicylic Acid
SAs	Sulfonamides
SE	Solvent Extraction
SLM	Supported Liquid Membranes
SMX	Sulfamethoxazole
SPE	Solid-Phase Extraction
SPME	Solid-Phase Microextraction
SPY	Sulfapyridine
STZ	Sulfathiazole
TCA	Tricyclic Antidepressants
TC	Tetracycline
TPB	Triphenylbenzene
TRIP	Triptycene
